# Supplementary material for: Expression of Nerve Growth Factor and Its Receptor TrkA in the Reproductive System of Adult Zebrafish
Source: Vet Sci. 2022 May 6;9(5):225. doi: 10.3390/vetsci9050225 (PMC9144415; doi:10.3390/vetsci9050225)
Supplement: Supplementary file 1 [file vetsci-09-00225-s001.zip › vetsci-1694606-supplementary.pdf]

veterinary sciences

MDPI

Supplementary Figure S1

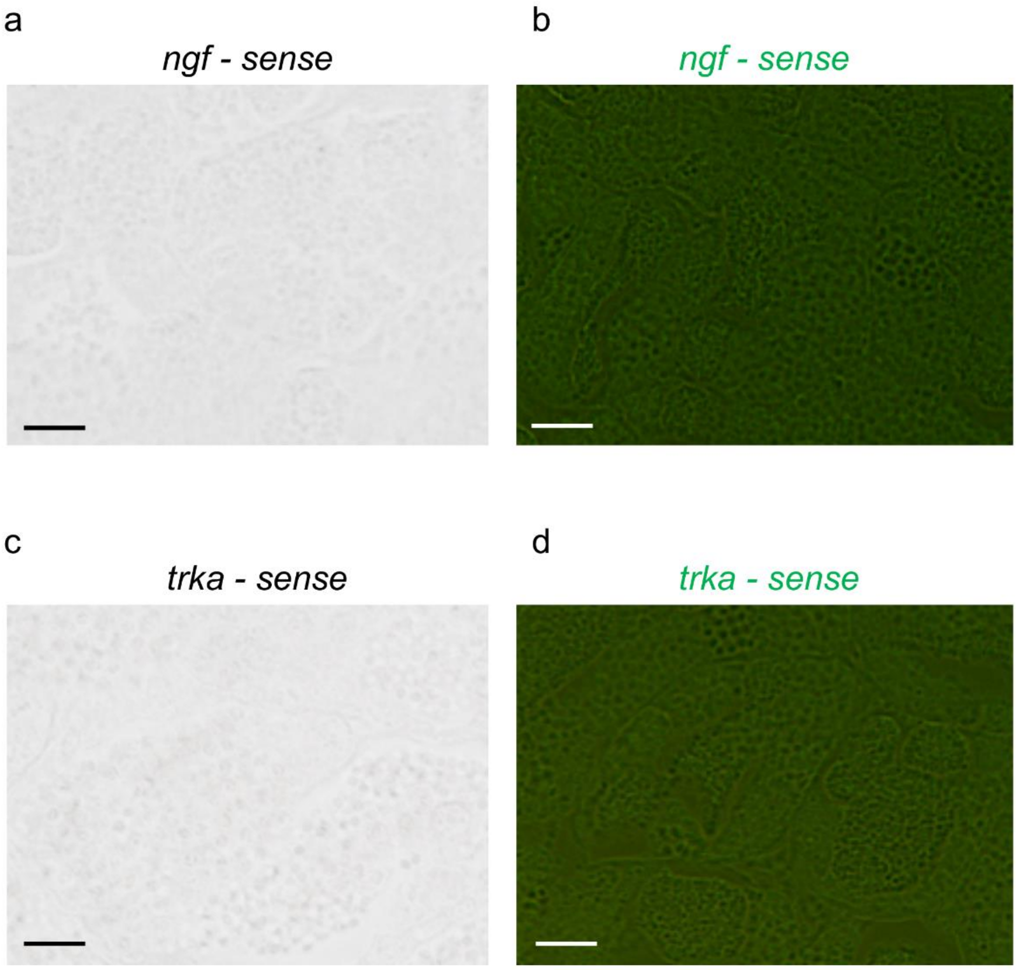

Supplementary Figure S1. ngf and trks sense probes staining in testi. (**a**–**b**) ngf sense probe, choromogenic and fluorescence in situ hybridization stainings. (**c**–**d**) trka sense probe, choromogenic and fluorescence in situ hybridization stainings, on paraffin sections of adult zebrafish testis.

Supplementary Figure S2

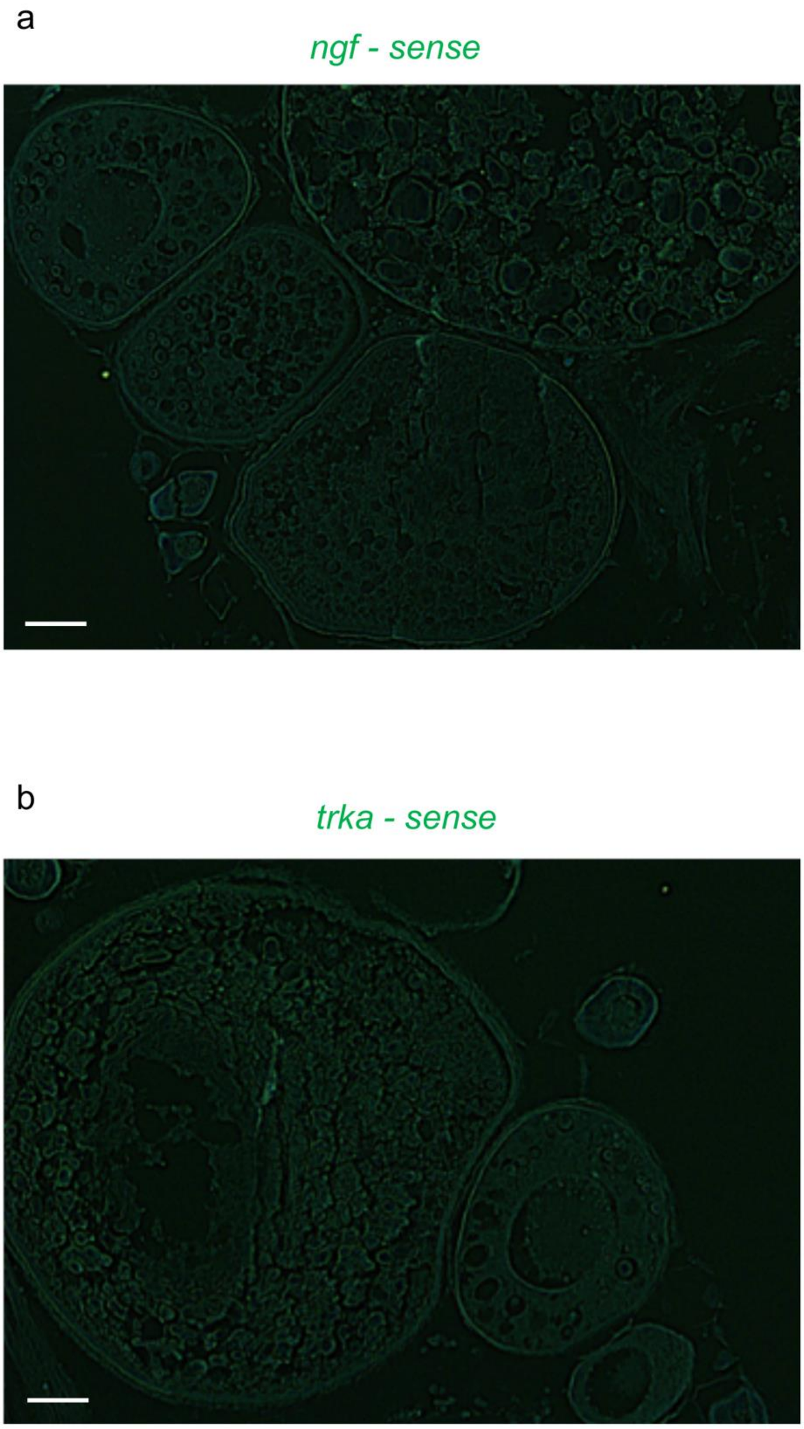

Supplementary Figure S2. *ngf* and *trka* sense probe staining in ovary. (**a**–**b**) ngf and trka sense probes, choromogenic and fluorescence in situ hybridization stainings on paraffin sections of adult zebrafish ovary.
